# Networks of random trees as a model of neuronal connectivity

**DOI:** 10.1007/s00285-019-01406-8

**Published:** 2019-07-24

**Authors:** Fioralba Ajazi, Valérie Chavez–Demoulin, Tatyana Turova

**Affiliations:** 1 Department of Mathematical Statistics, Faculty of Science, Lund University, Sölvegatan 18, 22100 Lund, Sweden; 2 Faculty of Business and Economics, University of Lausanne, 1015 Lausanne, Switzerland; 3 IMPB - The Branch of Keldysh Institute of Applied Mathematics of Russian Academy of Sciences, Moscow, Russia

**Keywords:** Neuronal network, Branching process, Random graph, 94C15, 92B20, 62P10

## Abstract

We provide an analysis of a randomly grown 2-d network which models the morphological growth of dendritic and axonal arbors. From the stochastic geometry of this model we derive a dynamic graph of potential synaptic connections. We estimate standard network parameters such as degree distribution, average shortest path length and clustering coefficient, considering all these parameters as functions of time. Our results show that even a simple model with just a few parameters is capable of representing a wide spectra of architecture, capturing properties of well-known models, such as random graphs or small world networks, depending on the time of the network development. The introduced model allows not only rather straightforward simulations but it is also amenable to a rigorous analysis. This provides a base for further study of formation of synaptic connections on such networks and their dynamics due to plasticity.

## Introduction

A strong believe that a comprehensive knowledge of the wiring diagram of the entire brain is fundamental for understanding processing of information inspired laborious creation of datasets of connectivity on different levels: Allen Mouse Brain Connectivity Atlas (Oh et al. 2014) and Human Connectome Project (Van Essen et al. 2013), meso-scale, or Human Brain Project (Markram 2015), cell-level.

The effect of brain structure on brain performance can only be addressed in the context of dynamical processes on networks. As data in vivo (Oh et al. 2014), or even from the prepared *slices* (Perin et al. 2011) are enormously expensive, they can be complemented by the data obtained by simulated network growth. One of the well developed simulators of neuronal growth is NETMORPH (Koene et al. 2009), which originates in earlier neurite growth models of van Pelt and Uylings (2002) and Uylings and van Pelt (2002).

Lately some progress has been made to quantify the relations between structure and functions, which is a very delicate task when it concerns changes only on a micro-scale, such as synaptic connections. Examples of this include; Mäki-Marttunen et al. (2013) defined, with a help of graph-theoretic measures, the aspects of structure which have the greatest effect on the network excitability. Borisyuk et al. (2014) introduced and studied a model of a brain development which is capable of exhibiting intimate relations between structure and a complex function of an entire organism, such as motion. Recently it has been reported (Guzman et al. 2016) that changes in micro-connectivity, that is, on the synaptic level, also contribute to efficient memory storage and retrieval in hippocampal networks. Some relevant discussion on modelling brain oscillations on random graphs is provided by Kozma and Puljic (2015).

Nearly all contemporary analyses of empirical data on the neuronal networks appeal to the graph theory for their very relevant terminology and methodology. Viewing neuronal networks as abstract graphs results in numerous attempts to classify these networks using their empirical or statistical characteristics, such as for example, degree, clustering coefficient or the shortest path (Bullmore and Sporns 2009). Since there is only a few different well-studied graph models, this classification remains rather rough as it divides all networks into large classes most often referring to Erdős and Rényi (1960) random graphs, regular random graphs (Bollobás 2001), or the so-called “small world networks” popularized by Watts and Strogatz (1998). Furthermore, all these models miss the important characteristics, namely the dependence on space, that is, distance between the nodes, and the dependence on time [see discussion of Fornito et al. (2013)]. Graph models in metric spaces with polinomial decay of the probabilities of connections with respect to distance have been studied recently by Ajazi et al. (2017), Janson et al. (2015), Bringmann et al. (2019). Observe that all the above mentioned models of random graphs assume the independence of the edges. The latter can hardly be justified for a biological network.

Overall the limitation of applicability of popular random graph models to the study of complex biological networks is clear, and assigning the edge weights to the connections, in other words defining a proper graph model, remains the key challenge for neuromodelling [see also Fornito et al. (2013), Ferrario et al. (2018)]. We address this challenge here analyzing the dynamical graph model introduced by Ajazi et al. (2015), which is greatly inspired by the original work of van Pelt and Uylings (2002) and the study by Mäki-Marttunen et al. (2013).

Recall that van Pelt and Uylings (2002) model neurons by rooted binary trees. Their detailed description of the outgrowth of axonal and dendritic arborizations allows one to assess the parameters of the model in agreement with physiological data. A simulator NETMORPH helps to estimate neural connectivity (Mäki-Marttunen et al. 2011; van Pelt and van Ooyen 2013; McAssey et al. 2014) using the following observation. The neurons communicate through synaptic connections which require physical contact between axons and dendrites of two corresponding neurons. Hence, it is suggested by the work of Peters and Feldman (1976) that the probability of synaptic connection between two neurons is proportional to the overlap area of the corresponding axonal and dendritic arborizations of these neurons. Hence, results of van Pelt and van Ooyen (2013), McAssey et al. (2014), Aćimović et al. (2015), and even earlier ones by Stepanyants and Chklovskii (2005) provide the estimates for the *potential* synaptic connectivity only. It is also recognized, however, that the distance is not the ultimate parameter, and hence the proximity of the axons and dendrites provides only the necessary condition for the synaptic contact [see also a critical review of the related works in Rees et al. (2017)].

The advantage of a relatively simple model by Ajazi et al. (2015) is that it is amenable to a rigorous analysis. On the other hand this model is compatible statistically with previously studied models. For example, the results on dendritic mass distributions as functions of Euclidean distance reported by Ajazi et al. (2015) (Fig. 5) are very similar to those by van Pelt and van Ooyen (2013) (Fig. 13) obtained with the help of NETMORPH (Koene et al. 2009).

The novelty here is that we study the main characteristics of the network (the frequency of connection, the degree distribution, the shortest path and the clustering coefficient) as functions of time. It has already been observed in Ajazi et al. (2015) that a growing network undergoes phase transitions in structure due to evolution in time. Here we provide further evidence of that with statistical analysis. We show that the tuning of parameters results in structural changes in the model: the model may possess properties closed, for example, to those of a classic random graph, or to the ones of a “small world” network.

The most common approach to estimate the probabilities of connection in related morphological networks (van Pelt and van Ooyen 2013; McAssey et al. 2014; Aćimović et al. 2015) treats the areas of the dense dendritic mass by the convex sets, for example, balls. However, it has been disclosed by Ajazi et al. (2015) that an accurate consideration of spatial trees of connections (which are not convex subsets of the plane) results in a more subtle dependence of the connections on the Euclidean distances between the neurons rather than commonly assumed polynomial or exponential decay. We elaborate this approach here and study the probabilities of connections as functions of both space and time, and of the branching parameter. Although we still cannot perform the entire rigorous analysis of our model, we can derive some exact equations which allow us to come up with at least qualitative conclusions and hypotheses which we test statistically.

Our study reveals the properties of the network as functions of several parameters (time, distance and branching), which does not seem to be a realistic task for complex models with many more parameters.

We also address the question of scalings between parameters, which can be clarified only analytically, but which has a strong impact on the modelling. Even if the size of the neuronal network is finite it is definitely large, and therefore it might be useful to consider limits when the number of nodes (a large parameter) goes to infinity, in which case all the remaining parameters (small ones as well) must be scaled correspondingly. Using data on physical sizes in a cortex [for example, Rolls (2016)] one can fit our model into a real scale of biological network.

Finally, as in the previously cited works starting with van Pelt and Uylings (2002), we study here the formation of only *potential* connections in the network. Our results provides a means to assess the parameters to model a graph with some desired properties (as for example, degree distribution, connection probabilities of a given strength, high or low clustering coefficients etc.) which provides an underlying structure for a more elaborated model of actual connections. Then the actual synaptic connections can be modelled as a way of pruning the given potential connections according to some biologically justified rule. The dynamics of synaptic connections due to plasticity should be then modelled as another process on the underlying structure. This all prompts the development of models of percolation processes on random graphs with dependencies between the edges.

## Model

### Biological interpretation

Recall the definition of a directed random growing network given by Ajazi et al. (2015).

We assume that we are given a finite set of points *V* on a plane, where each $v\in V$ represents a location of one neuron. Each neuron develops its axonal tree independently of other neurons. The dynamics of the trees will be defined later by two independent processes of elongation and branching. Notice that for a simplicity we consider only axonal trees. To account for the dendrites we set a ball of radius *r* around each point *v* to represent the physical size of the soma together with an area of dense dendrites of the neuron at *v*. A model with dendritic arborization can be treated in a similar way, but this we leave for later studies.

Observe that this model of connectivity is similar to that of Borisyuk et al. (2014), where the axons are modelled as trajectories of stochastic (but not branching as in our case) processes, while the dendrites are also considerably simplified.

### The associated graph model

Assume that *V* has a Poisson distribution on a set $\Lambda =[-w,w]\times [-w,w]$. with some intensity $\mu$. Hence, on the average there are $(2 w)^2\mu$ nodes in $\Lambda$.

Let $t\ge 0$ denote time. First we describe the dynamics of a tree $\mathbf{T}_v(t)$, $v \in V$. It is given by the following branching random walk on a plane. Set $\mathbf{T}_v(0)=v.$ From a point *v* a segment, or better a ray, as we shall consider directed graphs, starts to grow at time $t=0$ in a randomly chosen direction at a constant speed. We set the speed at 1, which is not a restriction, since the time can be rescaled. This initial ray splits at some random moment into two rays. The time of splitting is exponentially distributed with mean $1/\lambda$. Each of the two new rays develops independently in the same manner, but the directions of the new branches are independent random variables uniformly distributed on $[-\alpha , \alpha ]$, where parameter $\alpha \in [0,\pi ]$ represents the highest deviation from the direction of the splitting ray. This means that each of the rays independently chooses a random direction (within $\alpha$) to grow, and then each splits independently with the same intensity $\lambda$. Denote $\mathbf{T}_v(t)$ the resulting tree at time *t*.

Notice that graph $\mathbf{T}_v(t)$ is a subset of $\mathbf{R}^2$, that is, might grow outside of area $\Lambda$, while $v \in V \subset \Lambda \subset \mathbf{R}^2$, and $\mathbf{T}_v(t)$ as a graph on a plane might have self-intersections. We discuss the resulting boundary effect below.

Parameter $\alpha <\pi$ implies some memory of direction in the model: the smaller is $\alpha$, the stronger is the memory of direction. In particular, the $\alpha =0$ case is equivalent to the no branching case $\lambda =0$ when the constructed tree consists simply of one growing ray at any time *t*.

Consider now a collection of independent identically distributed trees $\left( \mathbf{T}_v(t), v\in V \right)$. We define a tree-distance $\rho$ from any *u* to any $v \in V$ as the smallest Euclidean distance ($\Vert \cdot \Vert$) between *u* and the tree $\mathbf{T}_v(t)$ as follows

$$\begin{aligned} \rho (u, \mathbf{T}_v(t)):=\min \{\Vert x-u\Vert :x\in \mathbf{T}_v(t)\}. \end{aligned}$$

#### Definition 1

We say that a neuron $v\in V$ has a connection to a neuron *u* at time *t* if the tree $\mathbf{T}_v(t)$ intersects a ball of radius $r>0$ with a center at *u*, or equivalently,

1 $$\begin{aligned} \rho (u, \mathbf{T}_v(t)) \le r. \end{aligned}$$

Finally, given a collection of independent identically distributed trees $\left\{ \mathbf{T}_v(t), v\in V \right\}$ define a directed graph *G*(*t*) on vertices *V* by setting an edge from $v\in V$ to $u\in V$ if $\rho (u, \mathbf{T}_v(t)) \le r$. Denote a probability of this edge by

2 $$\begin{aligned} p_{\lambda , \alpha }(t, u,v)=P\{\rho (u, \mathbf{T}_v(t)) \le r \}. \end{aligned}$$

Since every tree develops in the same manner we have due to the symmetry in the model

$$\begin{aligned} P\{\rho (u, \mathbf{T}_v(t)) \le r \}=P\{\rho (v, \mathbf{T}_u(t)) \le r \}, \end{aligned}$$

therefore the probability (2) depends only on the Euclidean distance $\Vert u-v\Vert$:

3 $$\begin{aligned} p_{\lambda , \alpha }(t, u,v)=p_{\lambda , \alpha }(t, \Vert u-v\Vert )=P\{\rho (u, \mathbf{T}_v(t)) \le r \}. \end{aligned}$$

Observe that only the edges outcoming from *different * nodes $v\in V$ in the directed graph *G*(*t*) are independent, whereas the edges outcoming from any given node $v\in V$ are not independent as they are constructed given the same tree $\mathbf{T}_v(t)$. Therefore probability (2) does not contain all the information on the distribution of the graph *G*(*t*). Nevertheless function $p_{\lambda , \alpha }(t, d)$ allows one to analytically derive the distributions for the local characteristics, such as the in-degrees and the out-degrees of the graph *G*(*t*).

It is possible to derive the following integral equation for the function $p_{\lambda , \alpha }(t,d)$ for all $\lambda \ge 0$ when $\alpha =\pi$. Let $q_{\lambda , \pi } ( t , d )=1-p_{\lambda , \pi }(t, d)$ for all $\lambda \ge 0$ when $\alpha =\pi$:

4 $$\begin{aligned} q_{\lambda , \pi } ( t , d )&= \int _{-\phi _0}^{+\phi _0} \int _0^{s_0(\phi )} \frac{\lambda e^{-\lambda s}}{2 \pi } (q_{\lambda , \pi } (t-s, {{\widetilde{d}}} (d,s,\phi ) ))^2 \mathrm {d} s \mathrm {d} \phi \nonumber \\&\quad + \int _{\phi _0}^{2\pi -\phi _0}\int _0^t \frac{\lambda e^{-\lambda s}}{2\pi } (q_{\lambda , \pi } (t-s,{\widetilde{d}}(d,s,\phi ) ))^2 \mathrm {d} s \mathrm {d} \phi \nonumber \\&\quad + \, e^{ - \lambda t } q_0( t , d ), \end{aligned}$$

where

$$\begin{aligned}&\phi _0= \arcsin \frac{r}{d}, \ s_0(\phi )=d \cos \phi -\sqrt{-d^2\sin ^2 \phi +r^2},\\&{\widetilde{d}}(d,s,\phi )= \sqrt{d^2 +s^2-2ds \cos \phi }, \end{aligned}$$

and

5 $$\begin{aligned} q_0(t,d)= {\left\{ \begin{array}{ll} 1 , &{}\quad \text { if } t < d - r,\\ 1- \frac{ 1 }{ \pi } \arccos { \frac{ d^2 + t^2 - r^2 }{ 2 t d } }, &{}\quad \text { if } t \in [ d - r , \sqrt{ d^2 - r^2 } ],\\ 1- \frac{ 1 }{ \pi } \arcsin { \frac{ r }{ d } } , &{}\quad \text { if } t > \sqrt{ d^2 - r^2 }. \end{array}\right. } \end{aligned}$$

This equation helps us to derive below some properties of the connection probabilities, even though the exact solution to it for general $\lambda >0$ remains an open problem. We treat the case $\lambda =0$ in detail below.

We study the main graph characteristics, such as degree distribution, minimum length path and the clustering coefficient of the simulated *G*(*t*) for different parameters $\alpha ,\lambda ,\mu , r, w$.

Keeping in mind the physical interpretation of these parameters we assume the following scaling

6 $$\begin{aligned} w= \Theta (1), \ \ r=o(1), \ \ \mu \gg 1, \ \ \mu r =o(1), \end{aligned}$$

where notation $\Theta (1)$ reads “of the order 1”.

### Advantages and limitations of the model

The introduced model is suitable both for simulations as well as for a rigorous analysis. It has a fair amount of parameters for biological interpretation on one hand, but still it is possible, in particular, for the probability of connections (4) to derive analytical dependence on all the involved parameters. Furthermore, function $p_{\lambda , \alpha }(t, d)$ allows one to derive analytically the distributions for the local characteristics, such as the in-degrees and the out-degrees of the graph *G*(*t*). This relation has not been disclosed in other studies of the related models of morphological growth.

The scaling (6) assumed above means that we take the area where the network grows as a unit, while the number of neurons is very high, and the axons are very thin. This assumption is in agreement with the biological data on cortex (Rolls 2016) that the dendrites of cells are typically in the region of 500 $\upmu$m in diameter, and their axons can be distributed in patches 200–300 $\upmu$m across, separated by distances of up to 1 mm (Martin 1984).

Ajazi et al. (2015) reported that the probability of average connectivity in the graph *G*(*t*) is not a monotone function of the distance between the nodes when $\alpha = \pi$. Below we empirically confirm this fact also for $\alpha < \pi$. Observe that although this contradicts a common assumption on the monotone decay of neuronal connections with the distance, the non-monotonicity of the strength of of connections between small groups of neurons as a function of their interspaces was already observed by measurements taken in Perin et al. (2011). Our results also contribute to the understanding of the so-called patchy connectivity in the cortex, studied for example, by Voges et al. (2010), where the dendrites are assumed to by centered in the soma while the center of mass of axonal fields is at some distance from the soma. The latter assumption is in agreement with our model.

A commonly adapted approximation of the axonal mass distribution by convex sets (in dimensions 2 or 3) leads to a sharp decay of the probabilities of connections with distance. On the other hand, our approach which takes into account that the tree on a plane is non-convex, provides an explanation for the formation of long-range connections. The latter is considered to be an important feature of a functional network by Voges et al. (2010).

We argue that graph *G*(*t*) undergoes structural transitions, because its characteristics exhibit different features depending on the time of the development. As a null hypothesis we use the assumption that the measurements are made on the classic random graph model *G*(*n*, *m*) with *n* vertices and *m* independent edges, where *n* and *m* equal, correspondingly, the number of vertices and edges in the considered *G*(*t*).

There are some natural questions that have to be addressed in future studies. We begin with the boundary conditions. In our model the neurons located at points close to the boundary of $\Lambda$ might grow axon trees beyond the area $\Lambda$, and all the trees are identically distributed. However closeness to the boundary affects the in- and out-degrees of the neurons; they become smaller (see analysis below).

One could study this model in a torus to avoid the boundary effects. However this choice is perhaps less realistic for the neuromodelling.

Some other scenarios could include:

(I) As soon as a branch of a tree touches the boundary of $\Lambda$ it stops growing.
(II) As soon as a branch of a tree touches the boundary of $\Lambda$ it continues to grow inside $\Lambda$ as a reflected motion.

The latter option (II) might be more realistic as it models the boundary as special conditions for the neurons. This may lead to a more dense distribution of the axons and dendrites at the boundary.

Finally we notice that considering a network on a plane makes sense as some of the brain tissue form 2-dimensional surfaces (Braitenberg and Schüz 1991). Secondly, and most importantly, one can also adapt our analysis for 3-dimensional growth. This will be a subject of future work. Observe here that in a tree grown in 3-dim with the same algorithm (except that the direction is chosen uniformly in 3-dim space) the self-intersections will happen with zero probability, which makes a model even more relevant. Notice that Eq. (4) is straightforward generalized for dimension 3. However, to make the same model tractable in dimension 3 as well one could incorporate some features typical for the brain area which is being modelled. In particular, one may consider axon growth which is not radially symmetric in dimension 3.

## The degree

We study the in- and out- degrees here. Our formula provide the functional dependence between the parameters of the model. In particular, we derive the conditions to have “hubs”, that is, vertices with a very high degree observed in real neuronal tissue (Van den Heuvel and Sporns 2013).

### The out-degree

The *out-degree*$\nu ^{out}_v(t)$ of a node *v* in a graph is the number of edges out-going from *v*. Hence, $\nu ^{out}_v(t)$ in our graph *G*(*t*) counts the number of points of the Poisson process on $\Lambda$ which are at a maximum distance *r* from the tree $\mathbf{T}_v(t)$.

Consider graph *G*(*t*) with a radial symmetry, assuming that $\alpha =\pi$.

Let $A_r(\mathbf{T}_v(t))$ denote the *r*-neighbourhood of $\mathbf{T}_v(t)$ in $\mathbf{R}^2$. For any set *B* in $\mathbf{R}^2$ let us denote |*B*| the area of this set. Then conditionally on a random parameter $\mu \left| A_r(\mathbf{T}_v(t)) \cap \Lambda \right|$ the out-degree of the neuron *v* is a (compound) Poisson-distributed random variable, that is

7 $$\begin{aligned} \nu ^{out}_v(t)\sim \text{ Po }(\mu \left| A_r(\mathbf{T}_v(t)) \cap \Lambda \right| ) \end{aligned}$$

(we use notation $\sim$ to denote equality in distribution here).

Notice that $\mathbf{T}_v(t)$ for any *t* is within the ball of radii *t* with a center at *v*. Therefore, to avoid the boundary effect, let us assume first that

8 $$\begin{aligned} t<w/2 \text{ and } |v|<w/2. \end{aligned}$$

Then we simply have

$$\begin{aligned} A_r(\mathbf{T}_v(t)) \cap \Lambda = A_r(\mathbf{T}_v(t)). \end{aligned}$$

When *r* is small, which is the case here, the area $\left| A_r(\mathbf{T}_v(t)) \right|$ is well approximated by

9 $$\begin{aligned} \left| A_r(\mathbf{T}_v(t)) \right| \approx 2(r+o(r))L_{v}(t), \end{aligned}$$

where $L_v(t)$ is the sum of all segments of tree $\mathbf{T}_v(t)$, and its distribution is derived by Ajazi et al. (2015). In particular,

10 $$\begin{aligned} \mathbf{E}{L_v( t)}= \frac{e^{\lambda t}-1}{ \lambda }, \end{aligned}$$

which is continuous at $\lambda =0$. Then it follows that

11 $$\begin{aligned} \mathbf{E} \nu ^{out}_v(t) = 2(r+o(r)) \mu \ \frac{e^{\lambda t}-1}{ \lambda } \end{aligned}$$

for all $\lambda \ge 0$ and $\alpha >0$. Assessing the parameters properly one can get any desired expected number of the connections in the model. To increase this number one should use $\lambda$ of order at least $|\ln (r\mu )|$.

When |*v*| is increasing, that is, as *v* is closer to the boundary its out-degree is decreasing, and the nodes at the corners of $\Lambda$ have the smallest expectation of the out-degree. However, due to the radial symmetry of the introduced tree evolution (recall that we consider case $\alpha = \pi$) even vertices at the corners will have the expected out-degree only 4 times less than the vertex at the origin. Hence, the boundary does not have a major effect on the order of the out-degree. Same argument holds for the in-degree as well.

It was proved by Ajazi et al. (2015) that in the case $\alpha =\pi$ the out-degree of $v=0$ in a graph *G*(*t*) with $t<w$ has an exponentially decaying tail. However, below we show that by tuning parameters one can get “hubs” as well in this model, and, moreover even in the case without branching. Recall that the existence of vertices-hubs is considered as remarkable feature of a neuronal network (Van den Heuvel and Sporns 2013).

### Conditions for the hubs

*No branching case*$\lambda =0$ is of a limited interest for modelling purposes, however, it provides some inside for the general one as it is exactly solvable and represents a marginal case for the model with a positive branching parameter. Furthermore, the feature of axons to grow in approximately straight lines unless their way is obstructed (i.e., as in our case when $\lambda =0$), was essentially used by Kaiser et al. (2009) to derive the exponential decay of the distribution of connection lengths.

When $\lambda =0$ the tree $\mathbf{T}_v(t)$ is simply a segment or a branch of length *t*, hence

12 $$\begin{aligned} \left| A_r(\mathbf{T}_v(t)) \right| =2rt+\pi r^2, \end{aligned}$$

and formula (7) yields

13 $$\begin{aligned} \nu ^{out}_v(t)\sim \text{ Po }\left( \left| A_r(\mathbf{T}_v(t) ) \right| \mu \right) \sim \text{ Po }\left( (2rt+\pi r^2)\mu \right) . \end{aligned}$$

This tells us that for all *t* as long as $rt\mu =o(1)$ the network consists mainly of disconnected nodes.

As $rt\mu$ grows, next phase of development of the network will be when the majority of the nodes still have the out-degree at most one. Then each a component of the network consists of a unique cycle with possible incoming trees at every node.

For all $r\le t\le w$, where *w* is at most of order constant (see (6)) all the trees-segments with high probability intersect at a positive angle. Therefore the area of the intersection of *r*-neighborhood of any tree with the *r*-neighborhood of all the remaining trees in the network is

$$\begin{aligned} \left| A_r\left( \mathbf{T}_u(t) \right) \cap \cup _{v\in V\setminus \{u\}} A_r\left( \mathbf{T}_v(t) \right) \right| = O\left( r \left| A_r( \mathbf{T}_u(t))\right| \right) =o\left( \left| A_r( \mathbf{T}_u(t))\right| \right) . \end{aligned}$$

Hence, the out-degrees defined by (13) can approximately be considered as independent since the respective areas of their trees have a very small overlap. Let $D_{max}(t)$ denote the maximal value among

$$\begin{aligned} |V|/4\sim \text{ Po } \left( \mu w^2 \right) \end{aligned}$$

*i*.*i*.*d*. copies of (13). Due to the properties of the Poisson distribution [consult also paper by Bollobás (1980)]

14 $$\begin{aligned} D_{max}(t)= & {} o_P(1), \text{ if } \mu rt \ll \frac{1}{\mu }, \end{aligned}$$

15 $$\begin{aligned} D_{max}(t)= & {} \Theta _P(1), \text{ if } \mu rt = \Theta \left( \frac{1}{\mu } \right) , \end{aligned}$$

and with a high probability

16 $$\begin{aligned} \frac{\log \mu }{\log \log \mu }\ll D_{max}(t) \ll \frac{\log \mu }{|\log (\mu rt )|}, \text{ if } \mu rt \gg \frac{1}{\mu }. \end{aligned}$$

For more details on the distribution of the maximum of the sequence of *i.i.d.* Poisson random variables, consult, for example, Anderson et al. (1997).

The last bound together with our assumptions (6) tells us that the range of parameters yielding “hubs” with high out-degree when $\lambda =0$ requires the following condition

17 $$\begin{aligned} \frac{1}{\mu ^2} \ll r\ll \frac{1}{\mu }. \end{aligned}$$

*Positive branching case*$\lambda >0$. Observe that given a set of vertices *V* the outdegrees for any $\lambda \ge 0$ are independent, since the trees are independent. Then it follows by approximation (9) that with a high probability the area of *r*-neighbourhood of a branching tree is of order $r e^{\lambda t}$, that is,

18 $$\begin{aligned} \left| A_r(\mathbf{T}_v(t)) \right| =O\left( r e^{\lambda t} \right) . \end{aligned}$$

Therefore when

19 $$\begin{aligned} \mu r e^{\lambda t}=\Theta (1), \end{aligned}$$

that is, of order constant, the area of intersections of the *r*-neighbourhood of all trees is *o*(1) due to $r=o(1)$. Hence, the subsets of *V* covered by *r*-neighbourhood of different trees can approximately be thought of as independent. Assumptions (6) yield $\mu r e^{\lambda t}\gg 1/\mu$, and similar to (16) we get that with a high probability

20 $$\begin{aligned} \frac{\log \mu }{\log \log \mu } \ll D_{max}(t) \ll \log \mu . \end{aligned}$$

where $\mu$ depends on *r* and $\lambda$ due to the relation (19).

### The in-degree

The *in-degree*$\nu ^{in}_v(t)$ of a node *v* in graph *G*(*t*) is the number of in-coming to *v* edges.

While formula (7) for the out-degree distribution does not use the connection probabilities, to compute the in-degree $\nu ^{in}_v(t)$ we shall make use of these probabilities. By the definition of edges in *G*(*t*) (see (1)) we have

21 $$\begin{aligned} \nu ^{in}_v(t)=\sum _{u\in V\setminus \{v\}}{} \mathbf{{1}}_{\{\rho (v, \mathbf{T}_u(t)) \le r\} } {\mathop {=}\limits ^{d}} \sum _{u\in V\setminus \{v\}} X_u(t), \end{aligned}$$

where, for any *t* and different *u*, the random variables $X_u(t)$ are independent and each has Bernoulli distribution with parameter $p_{\lambda , \alpha }(t, \Vert u-v\Vert )$ defined in (3).

Observe that the last formula suggests that (at least for some parameters) the distribution of the in-degree can be well-approximated by the Poisson distribution. Indeed, our numerical results confirm, that the in-degree is similar to the degree in the classical random graph, while the out-degree is different.

Equation (4) which defines (3) in the case $\lambda >0$ indicates an intricate dependence for the probability of connections $p_{\lambda , \alpha }(t, \Vert u-v\Vert )$ on the distance between the nodes (contrary to commonly assumed monotone polynomial or exponential decay) and on the time. It is monotone increasing in *t* and decreasing in $|u-v|$. Furthermore, there are certain time intervals when probability as a function of the distance, decays very slow on some intervals of values. This results in more similarities with the *G*(*n*, *m*) graph.

*No branching case*$\lambda =0$. Note that by (5) the probability $p_{0}(t,d)$ that at time *t* there is a connection from a fixed neuron *v* to another one *u* at distance $d=\Vert u-v\Vert >r$ is given by

22 $$\begin{aligned} p_{0}(t,d)=1-q_0(t,d)={\left\{ \begin{array}{ll} 0 , &{}\quad \text { if } t < d - r,\\ \frac{ 1 }{ \pi } \arccos { \frac{ d^2 + t^2 - r^2 }{ 2 t d } }, &{}\quad \text { if } t \in [ d - r , \sqrt{ d^2 - r^2 } ] ,\\ \frac{ 1 }{ \pi } \arcsin { \frac{ r }{ d } } , &{}\quad \text { if } t > \sqrt{ d^2 - r^2 }. \end{array}\right. }\nonumber \\ \end{aligned}$$

It is clear that for any fixed $d>r$, the function $p_{0}(t,d)$ increases in *t*, reaches its maximum when $t=\sqrt{d^2-r^2}$, and then remains to be at this constant value which depends only on the ratio *r* / *d*. Hence, in the case $\lambda =0$ a polynomial decay of the probabilities of connections is justified.

To avoid boundary effects, assume, $r \ll t<w/3$ and consider the in-degree $\nu ^{in}_v(t)$ of the vertex $v=\mathbf{0}$ at the center of $\Lambda$. Fix $\varepsilon$ arbitrarily and define

23 $$\begin{aligned} V_n =\{ u\in V: (n-1)\varepsilon < \Vert u\Vert \le n\varepsilon \} . \end{aligned}$$

This gives us the following partition of the set of vertices *V*

$$\begin{aligned} V=\cup _{n\ge 1} V_n, \end{aligned}$$

where by the definition of the set *V* each of $V_n$ has also a Poisson distribution

24 $$\begin{aligned} |V_n| \sim \text{ Po }( \pi (2n-1)\varepsilon ^2 \mu ). \end{aligned}$$

This helps us to find the following stochastic bounds for the in-degree defined in (21):

25 $$\begin{aligned}&\sum _{n= 1} ^{\left[ \frac{t}{\varepsilon }\right] }\text{ Po }\left( \pi (2n-1)\varepsilon ^2 \mu \min _{(n-1)\varepsilon \le d\le n\varepsilon }p_0 (t,d) \right) \preceq \nu ^{in}_v(t)\nonumber \\&\quad \preceq \sum _{n= 1} ^{\left[ \frac{t}{\varepsilon }\right] +1}\text{ Po }\left( \pi (2n-1)\varepsilon ^2 \mu \max _{(n-1)\varepsilon \le d\le n\varepsilon }p_0 (t,d ) \right) . \end{aligned}$$

Let us now rewrite (22) as follows

26 $$\begin{aligned} p_{0}(t,d)={\left\{ \begin{array}{ll} 0 , &{}\quad \text { if } d> t+r ,\\ \frac{ 1 }{ \pi } \arccos { \frac{ d^2 + t^2 - r^2 }{ 2 t d } } , &{}\quad \text { if } \sqrt{t^2+r^2}\le d\le t+r ,\\ \frac{ 1 }{ \pi } \arcsin { \frac{ r }{ d } } , &{}\quad \text { if } r<d \le \sqrt{t^2+r^2}, \end{array}\right. } \end{aligned}$$

where $d>r$. Recall that *d* here represents the distance between the centers of soma of two neurons. Then we derive from (25) for $t+r<w$

27 $$\begin{aligned} \sum _{n= \left[ r/\varepsilon \right] } ^{\left[ \frac{\sqrt{t^2+r^2}}{\varepsilon }\right] -1}2n\varepsilon ^2 \mu \arcsin { \frac{ r }{ \varepsilon n } } \le \mathbf{E}\nu ^{in}_v(t) \le \sum _{n= \left[ r/\varepsilon \right] } ^{\left[ \frac{t+r}{\varepsilon }\right] +1}(2n-1)\varepsilon ^2 \mu \arcsin { \frac{ r }{ \varepsilon n } }. \end{aligned}$$

Passing to the limit $\varepsilon \rightarrow 0$ we get

28 $$\begin{aligned} \int _{r} ^{\sqrt{t^2+r^2}}2x\mu \arcsin { \frac{ r }{ x } }dx\le & {} \mathbf{E}\nu ^{in}_v(t) \le \int _{r} ^{t+r}2x\mu \arcsin { \frac{ r }{ x } }dx\nonumber \\= & {} \int _{r} ^{\sqrt{t^2+r^2}}2x\mu \arcsin { \frac{ r }{ x } }dx +O(r^2\mu ) = 2\mu rt +O(r^{3/2}\mu ),\nonumber \\ \end{aligned}$$

which together with (25) yields

29 $$\begin{aligned} \nu ^{in}_v(t)\sim \text{ Po }\left( 2\mu rt +O(r^{3/2}\mu ) \right) , \end{aligned}$$

that has the same asymptotic for small *r* as the out-degree (13).

Observe that despite the same value asymptotic, there is a major difference between the spacial distribution of the set of vertices contributing to the in-degree and of the set of vertices contributing to the out-degree of a vertex. Conditionally on the event that there is an edge from *v* to *u* the location of the remaining vertices to which *v* has an edge is restricted to the area close to the interval of length *t* which starts at *v* and passes through a ball of radii *r* around *u*. However, conditionally on the event that there is an edge from *u* to *v*, the locations of other vertices with edges to *v* are independent of the location of *u*.

Finally we observe that when $\lambda =0$ the network after time $t=2w$ no longer changes since as soon as the “axons” pass the size of the area $\Lambda$ they do not return to $\Lambda$.

**Fig. 1:**
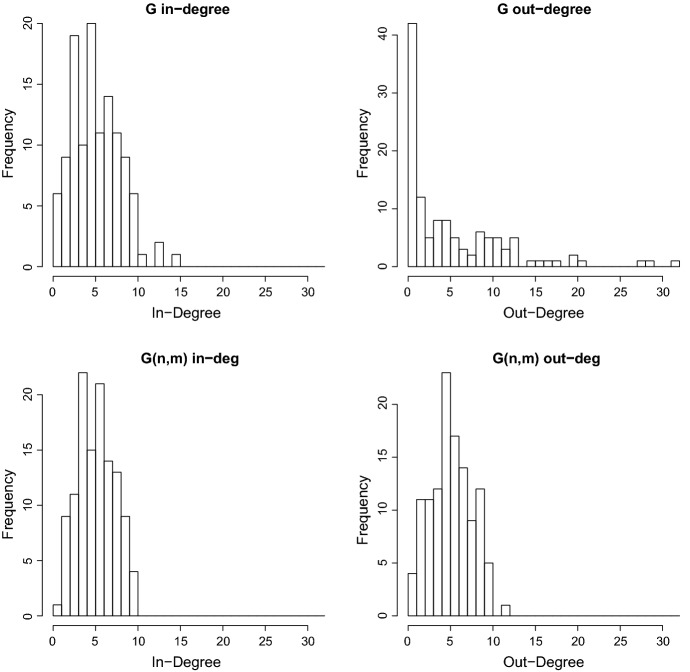
Computer simulations: Frequency of degree of the graphs *G*(*t*) and *G*(*n*, *m*) simulated with $\alpha =\pi /2$, $\lambda =1.5$, $\mu =100$, $T=1.5$, $w=1$

**Fig. 2:**
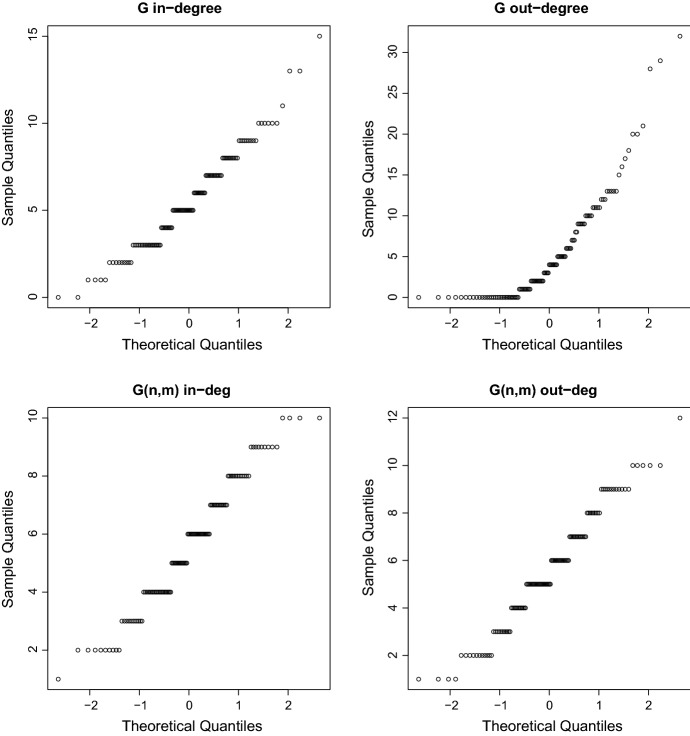
Computer simulations: Quantile-quantile plots against normal distribution for degree of the graphs *G*(*t*) and *G*(*n*, *m*) simulated with $\alpha =\pi /2$, $\lambda =1.5$, $\mu =100$, $T=1.5$, $w=1$

### Statistical analysis for the degree when $\lambda >0$

In this section, we run computer simulations generating networks with $\alpha =\pi /2$, $\lambda =1.5$, $\mu =100$ and empirically exploring the distributions of the in-degrees and out-degrees. Figure 1 represents histograms of in-degrees and out-degrees generated from one simulated network. One can observe that the maximal out-degrees exhibit the highest discrepancy between our model and the corresponding *G*(*n*, *m*) model, as we could already predict from our analysis. This is observed not only for this particular simulated network of Fig. 1 but for several other networks we generated. Another finding based on several simulations of such types of networks is that when $\lambda$ increases the differences between the in-degree distribution and the out-degree distribution vanish, similar to the classical random graph, where in- and out-degree have the same distribution. Going back to the in- and out-degrees distributions, we conduct a statistical analysis on the different degrees shown in Fig. 1, inferring which distribution fits the data best in each case. Figure 2 shows quantile-quantile plots of the different degrees data against the normal distribution. Although the discrete nature of the data appears in the plots, the normal distribution seems to adequately approximate the empirical distributions apart from the out-degree of *G*(*t*) (top right panel of Fig. 1). Table 1 provides the value of the Akaike criteria for each potential distribution fitted to the degrees. For each case, we retain the distribution which minimizes the AIC, that is the normal distribution for the in-degree of a *G*(*t*) graph, the exponential distribution for the out-degree of the *G*(*t*) graph and the Poisson distribution for the in and out-degrees of the *G*(*n*, *m*) graph. The estimated parameters values of the different distributions are shown in Table 2. Note that this inference is based on only one sample of trees generated from a Poisson distribution with intensity equal to 100. The normal distribution appears as the approximate limiting distribution of the Poisson. The exponential distribution fit is not surprising given the shape of the distribution of the out-degree of *G*(*t*) graph (cf. top right panel of Fig. 1). Note that some AIC values and estimated values are equal and correspond to the sample mean which is the same for the four types of degrees.

**Table 1:** Akaike criteria (AIC) corresponding to several candidate distributions fitted by maximum likelihood estimation for the four different degrees

Distribution	G in-degree	G out-degree	G(n,m) in-deg	G(n,m) out-deg
Normal	**590**.**77**	787.32	526.22	574.65
Poisson	592.02	1151.91	**524**.**33**	**549**.**66**
Exp	649.15	**649**.**15**	649.15	649.15
Gamma	NA	NA	529.08	558.17
Lognormal	NA	NA	540.12	574.65

The lowest AIC scores are highlighted in bold

**Table 2:** Estimated values of the parameters of the distribution which minimizes the AIC for the different degrees

G in-degree	G out-degree	G(n,m) in-deg	G(n,m) out-deg
Normal ($\mu ,\sigma$)	Exp($\lambda$)	Poisson($\mu$)	Poisson($\mu$)
${\hat{\mu }}=5.58 (0.26); {\hat{\sigma }}=2.85 (0.18)$	${\hat{\lambda }}=0.18 (0.016)$	${\hat{\mu }}=5.58 (0.22)$	${\hat{\mu }}=5.58 (0.22)$

The value in parenthesis is the standard error

## Statistical analysis of a network with branching $\lambda >0$

When $\lambda >0$ the axons within area $\Lambda$ may grow unbounded, or until the *r*-neighborhood around all the trees covers the entire area $\Lambda$. Hence, there is a time *T*(*r*) when the development stops due to the space limitation. Notice that $T(r) \rightarrow \infty$ if $r \rightarrow 0$, in which case the network becomes eventually fully connected.

Since the connectivity of our network as a function of time is monotone increasing for any given parameters, by the analogy with phase transitions in random graphs one may expect to find certain time intervals when the properties of network change significantly.

Below we provide the results of the simulated network dynamics to highlight the functions of different parameters of the network, particularly of time.

### Frequency of connection

It was observed by Ajazi et al. (2015) that the density in space of the axonal tree of one neuron is not monotone decreasing with the distance from this neuron. We expect this property to be reflected in the frequency of connections as well.

**Fig. 3:**
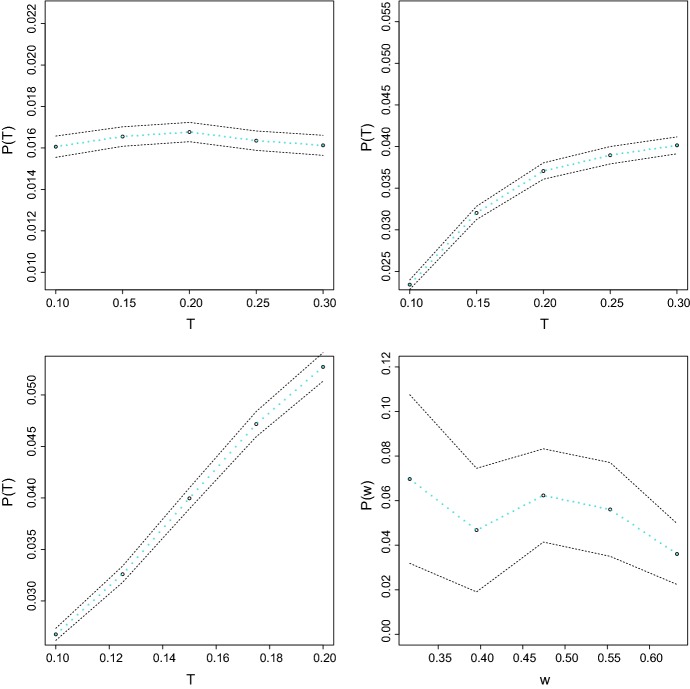
Computer simulations: Frequency *P*(*t*) with respect to *t* with $\alpha =\pi /2$, $t=[w,3w]$, for $\lambda =0$ (top left panel); $\lambda =5$ (top right panel); $\lambda =10$ (bottom left panel); Frequency *P*(*t*) with respect to distance *w*, for $\lambda =5$ given fixed time $t=3w$ (bottom right panel). The dotted curves are the 95% confidence intervals

**Fig. 4:**
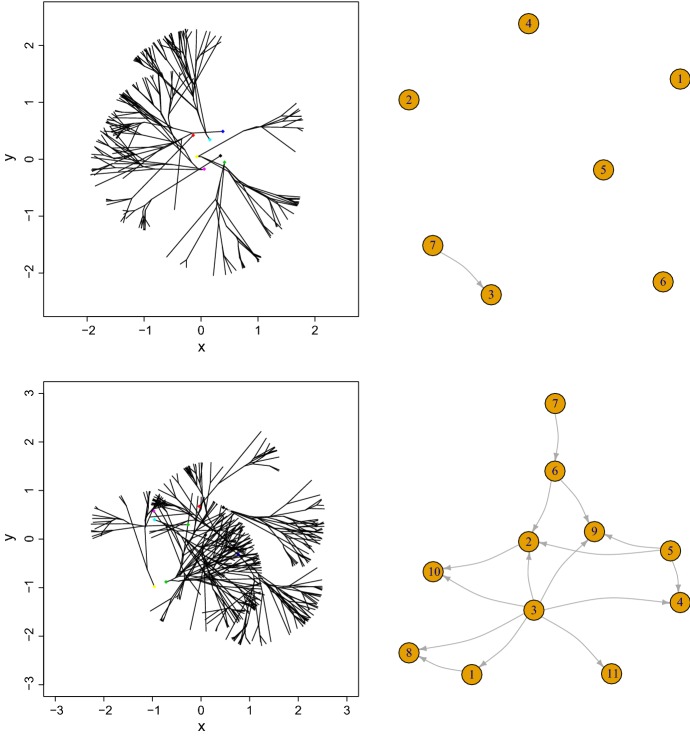
Computer simulations: Trees structures (left) with $\alpha =\pi /6$ and for $w=0.5$ (top), $w=1$ (bottom) and respective graph of connections *G*(*t*) (right)

**Fig. 5:**
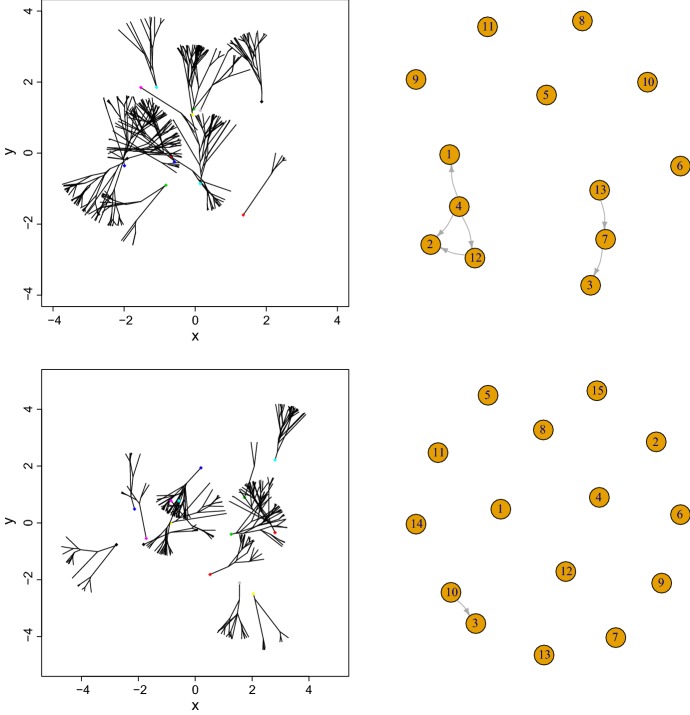
Computer simulations: Trees structures (left) with $\alpha =\pi /6$ and for $w=2$ (top), $w=3$ (bottom) and respective graph of connections *G*(*t*) (right)

Given $\mu =100$, $r=0.01$, $\alpha =\pi /2$ we generate 100 graphs, with different branching intensity $\lambda \in \{0,5,10\}$ and for different time moments *t* within the interval $[\sqrt{\mu }r, 3\sqrt{\mu }r]$. Furthermore, we consider different values for the size of the area, namely *w*. Keeping $\mu /w^2$, the intensity of the nodes per unit area being fixed, we simulate the model for various values of *w* which represents distances between vertices (bottom right panel of Figs. 3, 4 and 5).

We compute the frequency of connections *P*(*t*) in the graph for different values of the parameters $\mu$, $\mu /w^2$ and *t*, setting

$$\begin{aligned} P(t)=P_{w, \mu , r, \lambda , \alpha }(t) =\frac{\#\{ \text{ directed } \text{ edges } \text{ in } \text{ the } \text{ graph } \text{ G(t) } \} }{ \# \{ \text{ ordered } \text{ pairs } \text{ of } \text{ vertices } \text{ of } \text{ the } \text{ graph } \text{ G(t) } \} }. \end{aligned}$$

Hence, *P*(*t*) is an approximation for the graph probability $p_{\lambda , \alpha }(t, d)$ defined in (3). In particular, our simulated results for $\alpha =\pi$ also provide an approximation for the solution to the Eq. (4) averaged over the entire graph.

Figure 3 illustrates the growth of the frequencies of connections over time depending on the intensity of branching and on different distances *w*. Our simulations confirm the following properties of the frequencies

- *Top left panel* For $\lambda =0$ the number of connections is rather constant over time. This is also in a perfect agreement with formula (22) for the probabilities of connections: when *t* is of order *w* most of the vertices have their trees beyond area $\Lambda$, therefore probabilities of connections do not change for them after time *w*.
- *Top right panel* For small positive $\lambda =5$ the amount of connections increases over time and stabilizes after a certain time. The small $\lambda$ case deviates slightly from $\lambda =0$ (see Eq. 4), but again after the tree leaves the box only a few branches come back.
- *Bottom left panel* For a higher $\lambda =10$ the frequency of the probability grows linearly in time. High values of $\lambda$ imply that a tree grows almost as a ball with a bit shifted center from the soma. For a high $\lambda$ the probability of connections increases to one, and the frequency stabilizes after a certain time *T*(*r*) (not seen in the graph) as discussed above.
- *Bottom right panel* For fixed time $t=3w$ we compute the frequency *P*(*t*) for different distances *w*. We observe a non-monotonicity with respect to the distance.

Further investigations have shown that the frequency of connections is stable over time when $\lambda =0$ whaterver the values of $\alpha$ and that it increases before stabilization after a certain time around 0.2, for a positive $\lambda$.

Connectivity increases with time while it decays with the parameter *w*, which in our simulations measures the distance between nodes. Notice that the computations could have been performed directly using the exact coordinates of the nodes. But to simplify the procedure we introduce instead scaling of the space, i.e, the parameter *w*.

Figures 4 and 5, left panels exhibit the geometric characteristics of the network evolution for $$w\in \{0.5,1,2,3\}$$ respectively and report the corresponding connectivity (right panels) in the network. At the smallest distance, when $$w=0.5$$ there are fewer connections than when $$w\in [1,2]$$, while at $$w=1 $$ there is a maximum number of connections.

### Shortest path

Given an arbitrarily fixed set of parameters for our model, we now compute the average shortest path of *G*(*t*) and of the corresponding *G*(*n*, *m*), that is, where *n* and *m* are the same as for *G*(*t*).

Denote $d_{v\rightarrow u}$ the length of the shortest path from *v* to *u* in graph *G*, that is, the number of the edges it consists of. When there is no path from *v* to *u* we set $d_{v\rightarrow u}=0$. We also define the directed average shortest path length as follows

30 $$\begin{aligned} L({G})=\frac{1}{|V|}\sum _{v\in V}\frac{\sum _{u\in V: \ u\ne v}d_{v\rightarrow u} }{|V|-1}. \end{aligned}$$

**Fig. 6:**
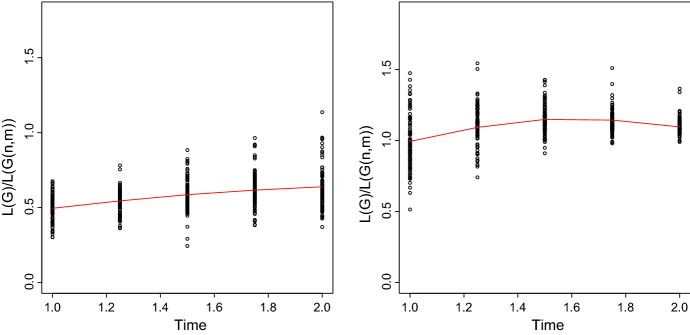
Computer simulations: Ratio between average shortest path of 100 simulations of *G*(*t*) and *G*(*n*, *m*) $\lambda = 1$, $r=0.1$, $w=1$ and $t=[1,2]$ for $\alpha =\pi /6$ (left panel); for $\alpha =\pi$ (right panel). For every $t\in [w,2w]$ we simulate 100 graphs and we report with the black cycles the ratio *L*(*G*(*t*)) / *L*(*G*(*n*, *m*)), the red line represents the mean value of this ratio (color figure online)

Figure 6 shows the ratio between the average shortest path of *G*(*t*) and the average shortest path of *G*(*n*, *m*) depending on the time *t* for $\alpha =\pi /6$ (left panel) and for $\alpha =\pi$ (right panel). It seems that the higher $\alpha$ the closer the ratio *L*(*G*(*t*)) / *L*(*G*(*n*, *m*)) is to one. Further investigations have shown that this ratio slowly tends to one for any value of $\alpha$ as the time increases. For both values of $\alpha$ the ratio remains almost constant between 0.5 and 1.1 when the time is big enough.

**Fig. 7:**
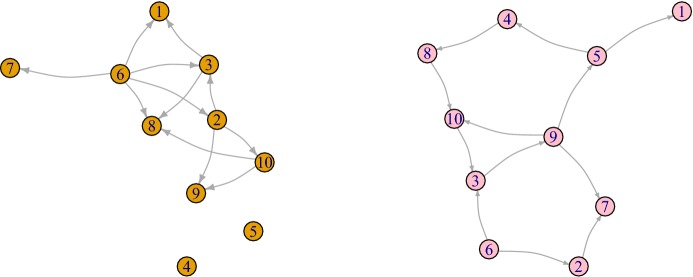
Computer simulations: Given the graph *G*(*t*) (left panel) and *G*(*n*, *m*) (right panel) for chosen parameters $\alpha =\pi /4$, $\lambda =6$, $t=0.4$, $w=0.2$, $r=0.1$, we compute the respective clustering coefficients $CC^{\rightarrow }(G)=0.19$ and $CC^{\rightarrow }(G(n,m))=0.041$

Our results show that when the time is small then the average shortest path length between any two nodes at distance bigger than *t* is lower in *G*(*t*) than in the corresponding *G*(*n*, *m*). This is due to the geometry of *G*(*t*) since here only a few short connections are available at small time, while *G*(*n*, *m*) the length of a path is not sensitive to the length of the edges. Further computer simulations have shown that for a wide range of parameters our model exhibits a low average shortest path, a property similar to the classic random graph (Bollobás 2001).

### Clustering coefficient

To study the clustering coefficient in the introduced directed graph we follow a common approach (for example, Fagiolo 2007). For any graph *G* on *V* vertices with adjacency matrix $(a_{uv})_{u,v \in V}$ the number of directed triangles $t_v^\rightarrow$ attached to the node $v\in V$ is given by the following equation

31 $$\begin{aligned} t_v^\rightarrow =\frac{1}{2}\sum _{w,u\in V}(a_{vw}+a_{wv})(a_{vu}+a_{uv})(a_{wu}+a_{uw}). \end{aligned}$$

Setting $\nu ^{tot}_v= \nu _v^{out}+\nu _v^{in}$, the directed clustering coefficient $CC^{\rightarrow }(G)$ for a graph *G* is defined as follows

32 $$\begin{aligned} CC^{\rightarrow }(G)=\frac{1}{|V|}\sum _{v\in V}\frac{t_v^\rightarrow }{\nu ^{tot}_v(\nu ^{tot}_v-1)-2\sum _{w\in V}a_{vw}a_{wv}}. \end{aligned}$$

Figure 7 provides an example of application of the last formula.

**Table 3:**
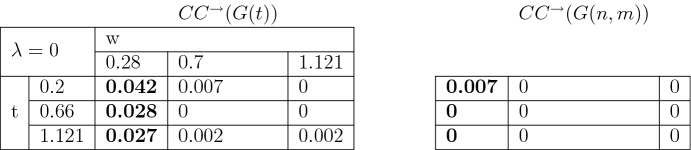
Clustering coefficients when $\lambda =0$. The values which are different with significance level 5 % are highlighted in bold

**Table 4:**
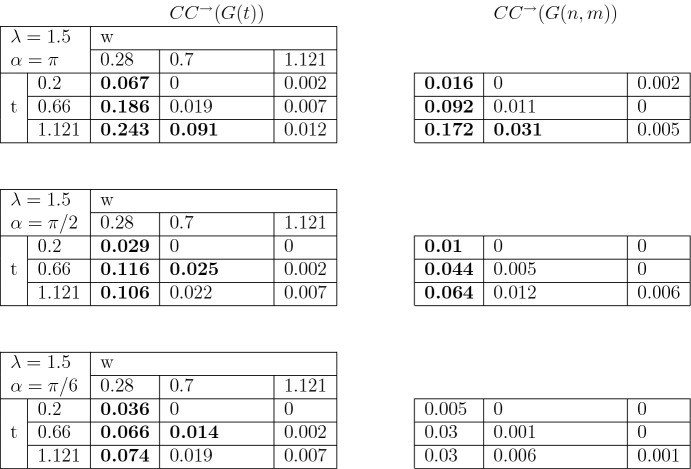
Clustering coefficients when $\lambda =1.5$. The values which are different with significance level 5 % are highlighted in bold

**Table 5:**
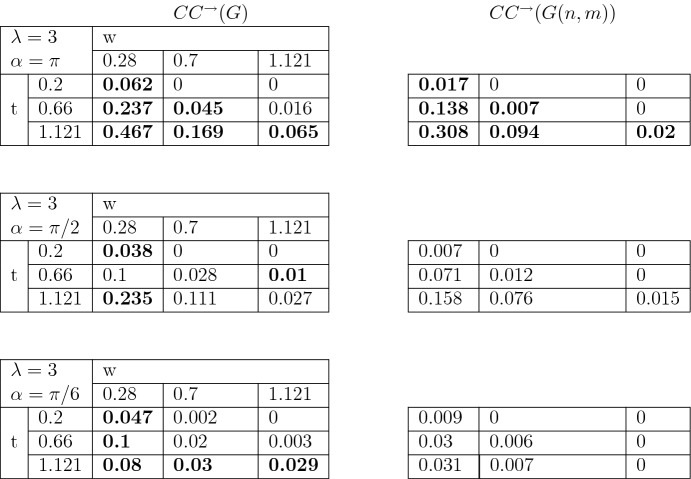
Clustering coefficients when $\lambda =3$. The values which are different with significance level 5 % are highlighted in bold

We further run 50 independent simulations of *G*(*t*) and of corresponding *G*(*n*, *m*) with $r=0.1$, $\mu =10$, with a range of parameters $\lambda \in \{0,1.5, 3\}$, $\alpha \in \{\pi , \pi /2,\pi /6 \}$, $t \in \{ 0.2, 0.66, 1.12\}$, $w\in \{0.28, 0.7, 1.121 \}$. Then we compute the clustering coefficients $CC^{\rightarrow }(G(t))$ and $CC^{\rightarrow }(G(n,m))$ for all possible combinations of these parameters. The results are given in Tables 3, 4 and 5. We highlight in bold those cases where $CC^{\rightarrow }(G(t))$ and $CC^{\rightarrow }(G(n,m))$ are different from each other at a significance level of 5%.

Our computations show that the clustering coefficient of *G*(*t*) is not monotone in time *t*. For the value $\lambda =0$ (Table 3) and a fixed distance $w=0.28$, $CC^{\rightarrow }(G(t))$ decreases in *t*. This fact is due to the geometry of the model. After a certain time the probability of forming single connections is higher than forming triples.

The non-monotone behavior in time can also be seen for the parameter $\lambda =1.5$, $\alpha =\pi /2$ and $\lambda =3$, $\alpha =\pi /6$, respectively, in Tables 4 and 5. The combination $(\lambda , \alpha )=(1.5, \pi /2)$ produces graphs where the tree structures have the shape of thigh cones due to the low frequency of branching. Also, despite the high intensity of branching, the pair $(\lambda , \alpha )=(3, \pi /6)$ produces as well a tree area very small due to the sharp angle of the directions of the trees growth.

On the other hand, when the trees grow more homogeneously, that is, high $\lambda$ and $\alpha =\pi$, we can observe that the clustering coefficient increases monotone with time.

We conclude that our model is capable to possess different properties depending on the parameters. In particular, our simulations prove a great variability of the clustering coefficient.

## Conclusions

We define a random graph *G*(*t*) which models potential synaptic connectivity between neurons. The network connectivity depends on different parameters which capture the most relevant features of potential synaptic development like intensity of branching, length, angle and speed of growth. We investigate the spatial development of those potential synapses, and show how degree distribution, frequency of connections, average shortest path and clustering coefficient evolve in time.

We show that the maximum of the in-degree of *G*(*t*) does not differ much from the one for the corresponding *G*(*n*, *m*) graph, unlike the maximum of the out-degree, which is much higher than the one in *G*(*n*, *m*).

Our results confirm that the frequency of connections increases monotone in time, but the highest value depends on the distances between the nodes.

Our simulations show for different parameters that our network depending on the parameters may resemble the typical characteristic of small world structure, that is, small average shortest path and high clustering coefficient, or it can be similar to the classic random graph model where both average shortest path and clustering coefficient are small.

Our study addresses also the question of scaling of the physical parameters to fit the model into real biological framework.

With this study we propose a model [simplified version of the one introduced by Mäki-Marttunen et al. (2013)], which is analytically tractable and allows simulations to mimic some properties of the real neural networks (Stepanyants and Chklovskii 2005).

The code which we used to produce the simulations is open under request.

Finally we remark that although here we consider 2-dimensional network instructed by the fact (Rolls 2016) that axonal trees form essentially 2-dimensional surfaces, our analysis is amenable for the models in dimension 3 as well (Goriachkin and Turova 2019). Let us also mention here that a related 3-dim model of cylinder percolation was studied by Tykesson and Windisch (2012).

The most challenging task remains to find a mathematically tractable model which is capable to quantify the relations between the structure on the cell level, as neuronal networks, and a macro behaviour, as, for example, movement. It is a subject of further study to apply our analysis to the model of Borisyuk et al. (2014).

Another direction to improve considered here model is to take into account both the axon and the dendritic arborization. Our approach should work in this case as well, however the analogue of Eq. (4) will be more involved.
